# Protein-Bound Uremic Toxins: New Culprits of Cardiovascular Events in Chronic Kidney Disease Patients

**DOI:** 10.3390/toxins6020665

**Published:** 2014-02-20

**Authors:** Shunsuke Ito, Masayuki Yoshida

**Affiliations:** 1 Life Science and Bioethics, Department of International Health Development, Tokyo Medical and Dental University, #953 M and D tower, 1-5-45, Yushima, Bunkyo-ku, Tokyo 113-8510, Japan; E-Mail: ito-shunsuke@kureha.co.jp; 2 Adsorptive Medicine Technology Center, KUREHA CORPORATION, 3-26-2, Hyakunin-cho, Shinjuku-ku, Tokyo 169-8503, Japan

**Keywords:** protein-bound uremic toxin, cardiorenal syndrome, indoxyl sulfate, *p*-cresyl sulfate, oral adsorbent

## Abstract

Chronic kidney disease (CKD) has been considered a major risk factor for cardiovascular diseases. Although great advances have recently been made in the pathophysiology and treatment of cardiovascular diseases, CKD remains a major global health problem. Moreover, the occurrence rates of cardiovascular events among CKD patients increase even in cases in which patients undergo hemodialysis, and the mechanisms underlying the so-called “cardiorenal syndrome” are not clearly understood. Recently, small-molecule uremic toxins have been associated with cardiovascular mortality in CKD and/or dialysis patients. These toxins range from small uncharged solutes to large protein-bound structures. In this review, we focused on protein-bound uremic toxins, such as indoxyl sulfate and *p*-cresyl sulfate, which are poorly removed by current dialysis techniques. Several studies have demonstrated that protein-bound uremic toxins, especially indoxyl sulfate, induce vascular inflammation, endothelial dysfunction, and vascular calcification, which may explain the relatively poor prognosis of CKD and dialysis patients. The aim of this review is to provide novel insights into the effects of indoxyl sulfate and *p*-cresyl sulfate on the pathogenesis of atherosclerosis.

## 1. Introduction

Cardiovascular death is approximately 10–20 times more frequent in dialysis patients than in healthy individuals [1], owing to a high prevalence of coronary artery diseases in this population [2]. Even in patients with mild-to-moderate chronic kidney disease (CKD), the estimated glomerular filtration rate (eGFR) is inversely correlated with the development of cardiovascular disease and mortality [3]. Keith *et al*. showed that in CKD patients, death occurred even before the patients had the opportunity to receive renal replacement therapy [4]. Thus, prevention of cardiovascular disease (CVD) is critical for patients with renal dysfunction. Traditional risk factors, such as age, diabetes, and hypertension, as well as non-traditional risk factors, such as uremic toxins, anemia, and Ca/P abnormalities, play central roles in the pathogenesis of atherosclerosis in patients with renal disease [5,6]. In spite of advances in therapeutic approaches for kidney complications, such as anemia and Ca/P imbalance, the therapeutic strategies aimed at reducing the amounts and effects of uremic toxins remain insufficient.

In 2012, the European Uremic Toxin Work Group (EUTOX) listed at least 88 uremic retention solutes [7]. Uremic toxins are divided into three major groups: small-molecular-weight water-soluble compounds, protein-bound compounds, and large-molecular-weight compounds [8]. In this review, we focused on protein-bound uremic toxins that are poorly removed by current dialysis techniques because of their size, which is larger than the pore size of dialysis membranes [9]. In particular, indoxyl sulfate and *p*-cresyl sulfate, which are considered representative protein-bound uremic toxins, are risk factors for CVD. Previous studies have suggested that these toxins are associated with the development of CVD and death in individuals with renal dysfunction.

The aim of this review is to provide novel insights into the effects of indoxyl sulfate and *p*-cresyl sulfate on the pathogenesis of atherosclerosis.

## 2. Formation of Indoxyl Sulfate and *p*-Cresyl Sulfate

Indoxyl sulfate is derived from tryptophan in dietary proteins. Tryptophan is metabolized to indole by the tryptophanase enzyme in the intestinal flora, such as *Escherichia coli*. Indole is carried to the liver through the portal vein, where it is converted into indoxyl, after which sulfate conjugation occurs. The resulting indoxyl sulfate is then excreted in the urine through the kidney. Therefore, serum levels of indoxyl sulfate increase when kidney function deteriorates [10,11].

The formation of *p*-cresyl sulfate is similar to that of indoxyl sulfate. In brief, tyrosine or phenylalanine is metabolized to 4-hydroxyphenylacetic acid and then decarboxylated to *p*-cresol, which undergoes sulfation by sulfotransferase to form *p*-cresyl sulfate. Serum concentrations of *p*-cresyl sulfate are also associated with GFR in pre-dialysis patients [12].

## 3. Clinical Studies Associating Protein-Bound Uremic Toxins and CVD

In dialysis patients, serum concentrations of indoxyl sulfate and *p*-cresyl sulfate are approximately 54 and 17 times higher, respectively, than the corresponding concentrations in healthy subjects [13]. Because these toxins bind to albumin, only about 30% of them are removed by hemodialysis. In contrast, over 60% of urea and creatinine are removed by hemodialysis [13]. Protein-bound uremic toxins have been associated with CVD. Barreto *et al*. demonstrated that high a serum indoxyl sulfate level was associated with death, including death due to cardiovascular events, in 139 patients with CKD. Serum indoxyl sulfate levels have also been shown to be correlated with vascular calcifications measured using multi-slice computed tomography (MSCT) or plain abdominal radiography [11]. Free (non-protein-bound), but not total, *p*-cresyl sulfate concentrations also seem to be associated with CVD and global mortality in both dialysis and pre-dialysis patients [14,15,16]. Further, free *p*-cresyl glucuronide, a metabolite formed by glucuronidation of *p*-cresol, was correlated with all-cause and cardiovascular mortality in patients with CKD [17,18]. These observations strongly suggest that these uremic toxins have causative roles in CVD.

## 4. Atherosclerosis under Uremic Condition

Atherosclerosis is an inflammatory vascular disease frequently associated with renal disease [19]. Atherosclerotic plaques are present in up to 30% of patients with CKD [20]. Endothelial dysfunction, especially leukocyte-endothelial interactions, plays an important role in the development of atherosclerosis [21]. Serum levels of inflammatory markers, such as C-reactive protein, interleukin-6 (IL)-6, IL-1β, tumor necrosis factor-α (TNF-α), and fibrinogen, are high in CKD patients [22,23]. Under these conditions, levels of cell adhesion molecules, such as intercellular adhesion molecule-1 (ICAM-1) and vascular cell adhesion molecule-1 (VCAM-1), along with those of E-selectin and P-selectin, are up-regulated in order to promote monocyte infiltration into the activated endothelium [21,24]. Migrated monocytes differentiate into macrophages and become fat-filled foam cells after phagocytosis of modified low-density lipoprotein (LDL) cholesterol, which leads to the formation of a fibrous cap, proliferation of vascular smooth muscle cells, and induction of angiogenesis, finally resulting in plaque rupture.

### 4.1. Effects of Protein-Bound Uremic Toxins on Endothelial Inflammation

Several reports have described that indoxyl sulfate induces ICAM-1 expression through the activation of nuclear factor-κB (NF-κB) and ROS production in cultured endothelial cells [25,26]. Pletinck *et al*. reported that injection of indoxyl sulfate to normal rats increased leukocyte adhesion to the vascular wall and their extravasation into venules [27]. These results suggest that indoxyl sulfate alone induces leukocyte adhesion to vascular endothelial cells.

**Figure 1:**
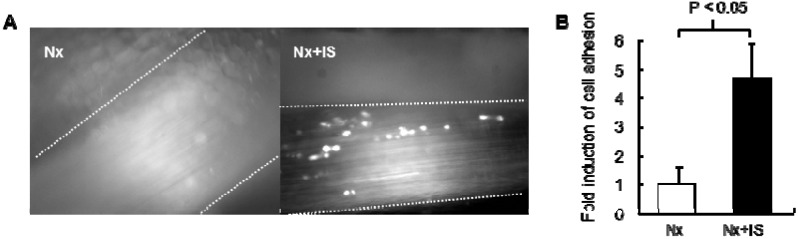
Effects of indoxyl sulfate on leukocyte-endothelial interactions in a mouse model of chronic kidney disease. (**A**) Representative snapshots from intravital video microscopic analysis of leukocyte adhesion in femoral arteries (vessel walls indicated by dashed lines) of 5/6 nephrectomized mice with (Nx+IS) or without (Nx) indoxyl sulfate treatment. White spots represent fluorescent leukocytes labeled using intravenously injected rhodamine 6G; (**B**) Quantitative analysis of leukocyte adhesion to femoral arteries. Data are expressed as mean ± SEM (N = 5).

**Figure 2:**
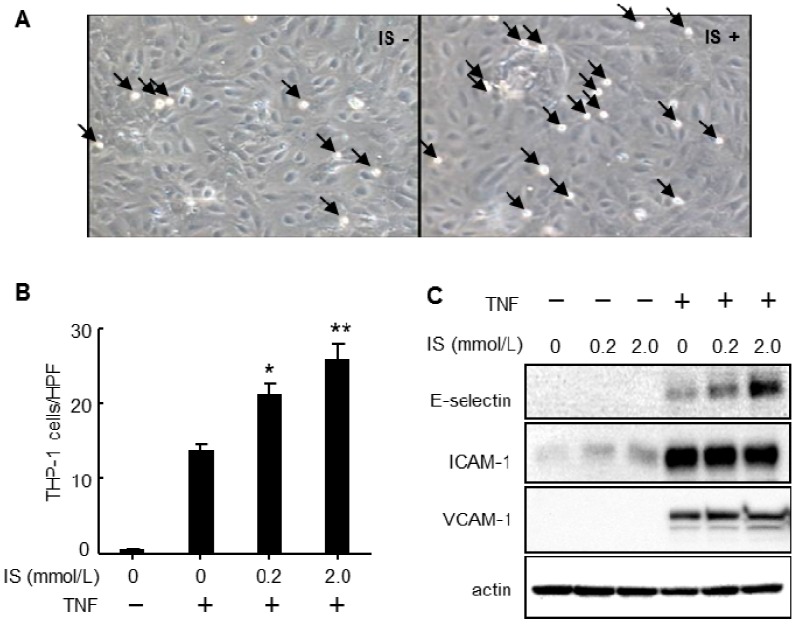
Effects of indoxyl sulfate on tumor necrosis factor-α (TNF-α)-induced leukocyte-endothelial interactions and expression of cell adhesion molecules. (**A**) Representative phase contrast micrographs showing adhesion of THP-1 cells to 100 pg/mL TNF-α-activated HUVECs with and without indoxyl sulfate (IS+ and IS−, respectively) pretreatment (2.0 mmol/L, 20 h). Arrowheads indicate adhered THP-1 cells (magnification, 200×); (**B**,**C**) Adhesion assay (**B**) and western blot analysis (**C**) of adhesion molecules in HUVECs treated with various concentrations of IS for 20 h, and then with (+) or without (−) TNF-α (100 pg/mL) for 4 h. Data from the adhesion assay are expressed as mean ± SEM (N = 10). * *P* < 0.01 *vs*. TNF (+) IS (−); ** *P* < 0.001 *vs*. TNF (+) IS (−). The data shown are representative of 3 independent experiments.

Because of the increased concentration of inflammatory cytokines in CKD patients, we hypothesized that indoxyl sulfate could enhance cytokine-induced leukocyte-endothelial interactions. Intravital microscopy revealed that administration of indoxyl sulfate to mice with subtotal nephrectomy induced leukocyte adhesion to the femoral artery (Figure 1). Pretreatment with indoxyl sulfate dramatically increased leukocyte adhesion and E-selectin expression in cultured endothelial cells after stimulation with TNF-α or IL-1β, although treatment with indoxyl sulfate alone did not affect leukocyte adhesion and E-selectin expression (Figure 2). Indoxyl sulfate also enhanced TNF-α-induced activation of NF-κB and c-jun N-terminal domain kinase (JNK) [26].

These observations suggest that indoxyl sulfate enhances vascular inflammation, and also provide insights into the mechanisms associated with higher cardiovascular mortality and death under inflammatory conditions in CKD patients [28]. Although oxidative stress-dependent pathways seemed to be involved, in similar conditions, angiotensin II, an oxidative stress inducer, did not increase cytokine-induced E-selectin expression. In addition to oxidative stress, other synergistic mechanisms might be involved in the effects of indoxyl sulfate.

Recently, indoxyl sulfate has been identified as a potent endogenous agonist for the human aryl hydrocarbon receptor (AhR), a member of the basic helix-loop-helix/Per-Arnt-Sim (bHLH/PAS) superfamily [29]. AhR mediates the toxic effects of numerous environmental contaminants, such as 2,3,7,8-tetrachlorodibenzo-*p*-dioxin (TCDD or dioxin). AhR has also been shown to mediate indoxyl sulfate-induced expression of monocyte chemoattractant protein-1 (MCP-1) in endothelial cells [30]. Further, both indoxyl sulfate and indole-3-acetic acid, which is also a metabolite of tryptophan, increase the expression of tissue factor, a key initiator of coagulation, in endothelial and mononuclear cells through AhR activation [31,32].

Indoxyl sulfate has been shown to induce endothelial cell senescence through p53 activation and ROS production [33]. It also induces junctional dispersal through the MEK-ERK-mediated phosphorylation of the myosin light chain kinase and myosin light chain [34]. Both *p*-cresyl sulfate and indoxyl sulfate inhibit endothelial cell proliferation and wound repair, and induce the release of endothelial microparticles, a marker of endothelial cell damage [35,36]. These observations suggest that endothelial dysfunction in CKD patients is, at least in part, caused by protein-bound uremic toxins such as indoxyl sulfate and *p*-cresyl sulfate.

**Figure 3:**
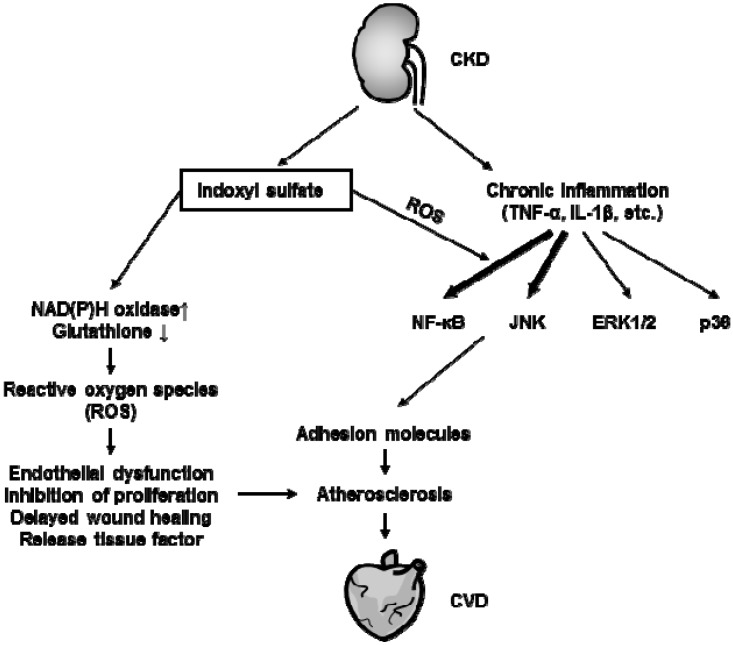
Effects of indoxyl sulfate on the vascular endothelium and cardiorenal syndrome. In renal failure conditions, levels of both pro-inflammatory cytokines and uremic toxins, such as indoxyl sulfate, are up-regulated. Indoxyl sulfate enhances cytokine-induced NF-κB and JNK signaling through oxidative stress-dependent pathways, resulting in increased expression of adhesion molecules. Indoxyl sulfate alone also mediates the release of reactive oxygen species (ROS) through activation of NAD(P)H oxidase and reduction in glutathione levels, leading to endothelial dysfunction, inhibition of proliferation, delayed wound healing, and release of tissue factor. These indoxyl sulfate-mediated toxic effects may lead to atherosclerosis resulting in a CVD, such as coronary artery disease.

Figure 3 summarizes the effects of indoxyl sulfate on vascular endothelial cells and the development of CVD. Accumulation of indoxyl sulfate *per se* contributes to the deterioration of endothelial function, and chronic inflammation further enhances endothelial inflammation, leading to atherosclerosis in CKD patients.

### 4.2. Effects of Protein-Bound Uremic Toxins on Oxidative Stress

Oxidative stress is created by the imbalance between the production and degradation of ROS. In uremic conditions, ROS inducers, such as NAD(P)H oxidase, xanthine oxidase, and myeloperoxidase, are activated, whereas levels of ROS scavengers, such as superoxide dismutase and glutathione, are down-regulated [23,37,38,39], causing high oxidative stress. Oxidative stress is a risk factor for not only CVD but also obesity, diabetes, hypertension, and dyslipidemia. Several oxidative stress markers have been shown to be inversely correlated with GFR [40]. 

Indoxyl sulfate induces ROS production in various cells, such as vascular endothelial cells, vascular smooth muscle cells, renal tubular cells, monocytes, and macrophages [26,33,41,42,43,44]. Activation of NAD(P)H oxidase and a decrease in glutathione levels have been proposed to mediate indoxyl sulfate-induced ROS production [42]. Fujii *et al*. reported that urinary indoxyl sulfate excretion was linearly associated with oxidative stress markers, such as 8-hydroxydeoxyguanosine and acrolein, in urine and in cardiac tissues [45]. We have also reported that the expression levels of p47*phox* and p22*phox*, 2 subunits of the NAD(P)H oxidase, increased in the aorta of nephrectomized mice administered indoxyl sulfate. Moreover, treatment with indoxyl sulfate increased ROS production and induced the translocation of p47*phox* to the membranes of monocytic THP-1 cells [46]. 

*p*-Cresyl sulfate also exhibits pro-oxidant properties in renal tubular cells by enhancing NAD(P)H oxidase activity. Administration of *p*-cresyl sulfate to nephrectomized mice caused oxidative stress, leading to renal tubular damage [47]. However, in vascular endothelial cells, *p*-cresyl sulfate had little effect on oxidative stress, compared to indoxyl sulfate [13]. Interestingly, other protein-bound uremic toxins, such as indoxyl glucuronide, an indole metabolite, *p*-cresyl glucuronide, a *p*-cresol metabolite, hippuric acid, and 3-carboxy-4-methyl-5-propyl-2-furanpropionic acid, have also been shown to induce ROS production in endothelial cells [13].

ROS also affect nitric oxide (NO) bioavailability, which contributes to vasorelaxation and inhibition of platelet aggregation, adhesion molecules, and proliferation of vascular smooth muscle cells [48]. Indoxyl sulfate decreased the endothelium-dependent vascular response in an *ex vivo* study [49], due to oxidative stress associated reduction of nitric oxide bioavailability [50].

### 4.3. Effects of Protein-Bound Uremic Toxins on Leukocyte Activation

In CKD patients, a compromised immune response increases the risks of CVDs and infections. Expression of Mac-1, a marker of leukocyte activation, and in particular, monocyte activation, was observed in CKD patients. Interestingly, functional inactivation of Mac-1, using an antibody or gene targeting, significantly reduced intimal hyperplasia in a mouse model of vascular inflammation. Moreover, we showed that an *in vivo* or *in vitro* treatment with indoxyl sulfate enhanced Mac-1 cell surface expression in monocytes and THP-1 cells, through pathways dependent on p38 mitogen-activated protein kinase (MAPK) and oxidative stress. Indoxyl sulfate also induces the mRNA expression of TNF-α, IL-1β, and IL-6 through p38 MAPK activation, and extracellular signal-regulated kinase (ERK), and NF-κB pathways in THP-1 cells [9]. Interestingly, Adesso *et al*. showed that indoxyl sulfate also increased the protein expression of inducible nitric oxide synthase (iNOS), cyclooxygenase-2 (COX-2), TNF-α, and IL-6 in lipopolysaccharide-stimulated macrophages, although indoxyl sulfate alone did not affect their expression [41]. Enhancement of NF-κB signaling might be involved in the additive effect of indoxyl sulfate. This synergistic effect is similar to that observed in vascular endothelial cells [26].

Protein-bound uremic toxins also affect T cell differentiation. Stimulation with *p*-cresyl sulfate of mouse splenocytes increased the percentage of Th2 cells [51], although it did not affect the percentages of CD8^+^ T cell subsets or regulatory T cells. Moreover, indoxyl sulfate induced Th17 cell differentiation via the c-Src or STAT-3 pathway, further worsening the autoimmune disease [52]. Therefore, whether protein-bound uremic toxins affect T cell function should be examined clinically.

### 4.4. Effects of Protein-Bound Uremic Toxins on Vascular Smooth Muscle Cells

Proliferation of vascular smooth muscle cells causes intimal hypertrophy, which contributes to the development of atherosclerosis [53]. Several reports have shown that indoxyl sulfate stimulates the proliferation of vascular smooth muscle cells through ROS-, ERK-, and/or p38 MAPK-dependent pathways [43,54,55]. It has been proposed that enhancement of platelet-derived growth factor (PDGF) expression might be involved in indoxyl sulfate-induced cell proliferation.

Vascular calcification is a strong predictor of cardiovascular mortality in individuals with renal insufficiencies [56]. In uremic conditions, bone metabolism markers, such as calcium/phosphate, parathyroid hormone, and vitamin D levels, become imbalanced, inducing hydroxyapatite (calcium phosphate crystals) accumulation in the vasculature. Further, differentiation of vascular smooth muscle cells into osteoblasts actively facilitates CKD-related vascular calcification. Adijiang *et al*. clearly demonstrated that administration of indoxyl sulfate promoted aortic calcification and wall thickening in hypertensive rats [57]. The trans-differentiation of vascular smooth muscle cells has been shown to involve osteoblast-specific proteins, such as core binding factor α-1 (Cbfa-1), osteopontin, osteocalcin, and alkaline phosphatase (ALP). Indoxyl sulfate may directly stimulate the differentiation demonstrated *in vitro* [58]. Both indoxyl sulfate and *p*-cresyl sulfate also induced de-differentiation in the kidney. These protein-bound uremic toxins have induced fibrosis via epithelial-to-mesenchymal transitions *in vivo* and *in vitro* [59,60,61].

## 5. Therapeutic Methods for Reducing Protein-Bound Uremic Toxins

The amounts of protein-bound uremic toxins can be reduced in the following two prominent ways: 

(1) removal from the blood and (2) reduction of precursors in the gut. (1) Because removal of protein-bound uremic toxins, such as indoxyl sulfate, depends on their diffusion rate, increasing the dialyzer mass transfer area coefficient and dialysate flow using two dialyzers in series is more effective for the removal of indoxyl sulfate and *p*-cresyl sulfate than conventional dialysis [62,63]. Furthermore, addition of sorbents, such as activated charcoal, to the dialysate enhances *in vitro* clearance [64]. However, unfortunately, these methods are not sufficient in terms of removal efficacy and are too expensive [9].

(2) The second approach is based on the reduction of indoxyl sulfate and *p*-cresyl sulfate metabolites. Serum concentrations of protein-bound toxins can be decreased by (i) reducing amino acid sources, by using a low-protein diet; (ii) preventing the generation of toxins or their precursors by using pre- or pro-biotics; and (iii) avoiding the absorption of toxins by using oral adsorbents. 

(i) A low-protein diet is easy to implement and an effective method, given that protein-bound uremic toxins, such as indoxyl sulfate and *p*-cresyl sulfate, are derived from dietary proteins. Interestingly, it has already been shown that serum indoxyl sulfate concentrations decreased in individuals receiving a low-protein diet [65]. However, a low-protein diet may lead to malnutrition, which may contribute to the poor outcomes in CKD patients. In fact, in the long-term follow-up Modification of Diet on Renal disease (MDRD) study, a very-low-protein diet did not delay the progression of kidney failure and appeared to increase the risk of death [66]. 

(ii) Since the intestinal flora is responsible for metabolizing amino acids to precursors of uremic toxins (e.g., tryptophan to indole), pre- and probiotics are considered potent agents for treatment against accumulation of uremic toxins. Several reports have shown that both pre- and probiotics could reduce uremic toxin concentrations in the serum or in fecal and urinary excretions [67,68,69,70,71,72]. However, the effects of these methods on the development of CKD or on a complication such as CVD have not yet been reported.

(iii) AST-120, an oral charcoal adsorbent, is known to reduce protein-bound uremic toxins, such as indoxyl sulfate and *p*-cresyl sulfate, through the absorption of their precursors, such as indole and *p*-cresol, respectively, in rodents and in humans including CKD patients. Many reports showed that AST-120 improved the estimated creatinine clearance and could delay the initiation of dialysis [73,74,75,76,77,78,79]. AST-120 may be effective for the treatment of vascular and cardiac diseases in CKD patients, and has been shown to attenuate the development of atherosclerosis through the suppression of TNF-α, IL-1β, and MCP-1 expression in the aorta of nephrectomized apolipoprotein E-deficient mice [80]. Treatment with AST-120 for 2 years significantly decreased the carotid intima-media thickness and arterial stiffness in non-diabetic CKD patients [81] compared to that in the non-treated group, whose condition worsened. AST-120 could also increase the flow-mediated endothelium-dependent vasodilatation, which is associated with a reduction in indoxyl sulfate [50]. AST-120 may have protective effects against cardiomyopathy in humans and rodents [9]. These observations suggest that AST-120 may represent an effective treatment for cardiorenal syndrome, although further studies, such as a prospective randomized controlled trial, are needed in order to clarify the cardiovascular protective effects of AST-120.

## 6. Conclusions

In this review, we focused on the effects of protein-bound uremic toxins, such as indoxyl sulfate and *p*-cresyl sulfate, on the development of atherosclerosis. Serum concentrations of protein-bound uremic toxins are associated with the incidence of CVD and mortality in patients with CKD, suggesting that these toxins are one of the CKD-specific non-traditional risk factors and play an important role in the development of cardiorenal syndrome. Several studies demonstrated that protein-bound uremic toxins have significant detrimental effects on vascular endothelial cells, smooth muscle cells, and leukocytes, including monocytes and macrophages. Oxidative stress and AhR seem to be involved in the development of those detrimental effects. Owing to the high protein-binding capacity of the toxins, therapeutic methods are limited. Currently, use of the oral sorbent, AST-120, is the only effective strategy for reducing the accumulation of protein-bound uremic toxins for preventing atherosclerosis in CKD patients.
